# Gonadotropin-inhibitory hormone action in the brain and pituitary

**DOI:** 10.3389/fendo.2012.00148

**Published:** 2012-11-28

**Authors:** Takayoshi Ubuka, You Lee Son, Yasuko Tobari, Kazuyoshi Tsutsui

**Affiliations:** Laboratory of Integrative Brain Sciences, Department of Biology, Center for Medical Life Science, Waseda UniversityTokyo, Japan

**Keywords:** gonadotropin-inhibitory hormone, gonadotropin-releasing hormone, gonadotropins, reproductive behavior, melatonin, stress, GPR147, RFamide-related peptide

## Abstract

Gonadotropin-inhibitory hormone (GnIH) was first identified in the Japanese quail as a hypothalamic neuropeptide inhibitor of gonadotropin secretion. Subsequent studies have shown that GnIH is present in the brains of birds including songbirds, and mammals including humans. The identified avian and mammalian GnIH peptides universally possess an LPXRFamide (X = L or Q) motif at their C-termini. Mammalian GnIH peptides are also designated as RFamide-related peptides from their structures. The receptor for GnIH is the G protein-coupled receptor 147 (GPR147), which is thought to be coupled to G_αi_ protein. Cell bodies of GnIH neurons are located in the paraventricular nucleus (PVN) in birds and the dorsomedial hypothalamic area (DMH) in mammals. GnIH neurons in the PVN or DMH project to the median eminence to control anterior pituitary function. GPR147 is expressed in the gonadotropes and GnIH suppresses synthesis and release of gonadotropins. It was further shown in immortalized mouse gonadotrope cell line (LβT2 cells) that GnIH inhibits gonadotropin-releasing hormone (GnRH) induced gonadotropin subunit gene transcriptions by inhibiting adenylate cyclase/cAMP/PKA-dependent ERK pathway. GnIH neurons also project to GnRH neurons in the preoptic area, and GnRH neurons express GPR147 in birds and mammals. Accordingly, GnIH may inhibit gonadotropin synthesis and release by decreasing the activity of GnRH neurons as well as directly acting on the gonadotropes. GnIH also inhibits reproductive behavior possibly by acting within the brain. GnIH expression is regulated by a nocturnal hormone melatonin and stress in birds and mammals. Accordingly, GnIH may play a role in translating environmental information to inhibit reproductive physiology and behavior of birds and mammals. Finally, GnIH has therapeutic potential in the treatment of reproductive cycle and hormone-dependent diseases, such as precocious puberty, endometriosis, uterine fibroids, and prostatic and breast cancers.

## INTRODUCTION

The decapeptide gonadotropin-releasing hormone (GnRH) is the primary factor responsible for the hypothalamic control of gonadotropin secretion. GnRH was first isolated from mammals (Matsuo et al., 1971; Burgus et al., 1972) and subsequently from birds (King and Millar, 1982; Miyamoto et al., 1982, 1984) and other vertebrates (for reviews, see Millar, 2003, 2005). Gonadal sex steroids and inhibin can modulate gonadotropin secretion. However, no hypothalamic neuropeptide inhibitor of gonadotropin secretion was known in vertebrates, although dopamine has been reported as an inhibitor of gonadotropin secretion in several fish groups. In 2000, a previously unidentified hypothalamic neuropeptide was shown to inhibit gonadotropin release from the cultured quail anterior pituitary gland and it was named gonadotropin-inhibitory hormone (GnIH; Tsutsui et al., 2000). Although it is now known that GnIH and its receptor are also expressed in the gonads of birds (Bentley et al., 2008; Maddineni et al., 2008; McGuire and Bentley, 2010; McGuire et al., 2011) and mammals (Zhao et al., 2010; Singh et al., 2011a,b; Li et al., 2012) including humans (Oishi et al., 2012), this review highlights the discovery of GnIH in the quail brain and the progress of GnIH research investigating its function in the brain and pituitary of birds and mammals. We also briefly review recent progresses in the study of GnIH peptides in fish and humans.

## DISCOVERY OF GnIH IN BIRDS

Gonadotropin-inhibitory hormone was first discovered in the brain of Japanese quail, *Coturnix japonica*, while searching a novel RFamide peptide in birds. RFamide peptides, which have an Arg-Phe-NH_2_ motif at its C-terminus, were first isolated in invertebrate species in the late 1970s. The first RFamide peptide, Phe-Met-Arg-Phe-NH_2_ (FMRFamide), was a cardioexcitatory molecule isolated from the ganglia of the venus clam *Macrocallista nimbosa* (Price and Greenberg, 1977). After the discovery of FMRFamide peptide, numerous RFamide peptides that act as neurotransmitters, neuromodulators, and peripheral hormones have been identified in various invertebrates, including cnidarians, nematodes, annelids, molluscs, and arthropods. Subsequent immunohistochemical studies in vertebrates suggested the presence of RFamide peptides in the central nervous system. It was revealed that some of the FMRFamide-immunoreactive (-ir) neurons projected close to the pituitary gland, suggesting a role in the regulation of pituitary function in vertebrates.

Tsutsui et al. (2000) have isolated a novel RFamide peptide from 500 brains of the Japanese quail by high-performance liquid chromatography (HPLC) and a competitive enzyme-linked immunosorbent assay using an antibody raised against the dipeptide Arg-Phe-NH_2_. The isolated peptide had a novel dodecapeptide structure, SIKPSAYLPLRFamide. Its C-terminus was identical to chicken LPLRFamide that was reported to be the first RFamide peptide isolated in vertebrates (Dockray et al., 1983), which is likely to be a degraded fragment of the dodecapeptide. The isolated peptide was localized in the hypothalamo-hypophyseal system, and shown to decrease gonadotropin release from cultured quail anterior pituitary glands (Tsutsui et al., 2000). This RFamide peptide was therefore named GnIH (Tsutsui et al., 2000).

**FIGURE 1:**
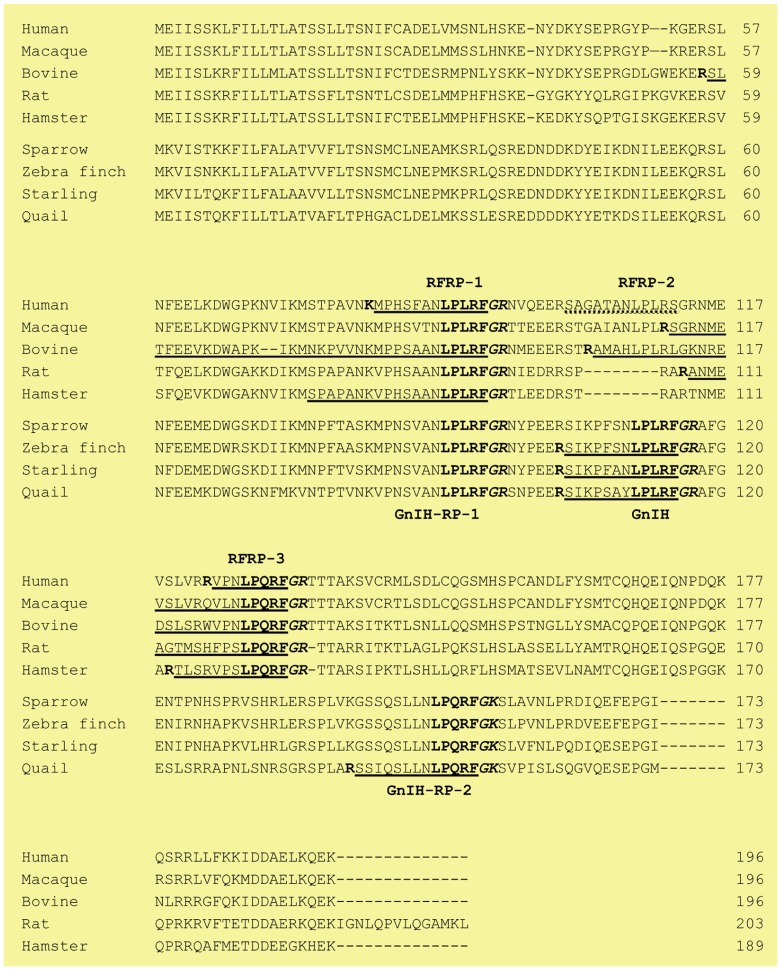
**The alignment of GnIH (RFRP) precursor polypeptides of birds and mammals**. The amino acid sequence of GnIH, GnIH-RP-1, GnIH-RP-2, RFRP-1, RFRP-3 with Gly (G) as an amidation signal and Arg (R) or Lys (K) as an endoproteolytic basic amino acid at the C-termini are shown in bold. Identified mature peptides are underlined. Endoproteolytic basic amino acid (R or K) at the N-termini are also shown in bold. A broken line indicates the putative human RFRP-2 sequence. Accession numbers are human (NM_022150), macaque (NM_001130827), bovine (NM_174168), rat (NM_023952), Siberian hamster (JF727837), white-crowned sparrow (AB128164), zebra finch (AB522971), European starling (EF486798) and Japanese quail (AB039815).

Following the isolation of GnIH in the quail brain, the precursor polypeptide for GnIH was determined (Satake et al., 2001). A cDNA that encoded GnIH precursor polypeptide was identified by a combination of 3′ and 5′ rapid amplification of cDNA ends (3′/5′ RACE; Satake et al., 2001). The GnIH precursor consisted of 173 amino acid residues that encoded one GnIH and two GnIH-related peptides (GnIH-RP-1 and GnIH-RP-2) possessing an LPXRFamide (X = L or Q) sequence at their C-termini (**Figure 1**). These peptide sequences were flanked by a glycine C-terminal amidation signal and a single basic amino acid on each end as an endoproteolytic site (**Figure 1**). GnIH-RP-2 was also identified as a mature peptide by mass spectrometry in quail (**Figure 1**; Satake et al., 2001). GnIH was further isolated as mature peptides in European starlings (Ubuka et al., 2008a) and zebra finch (Tobari et al., 2010) within the class of birds (**Figure 1**; for reviews, see Tsutsui and Ukena, 2006; Tsutsui et al., 2007, 2010a,b, 2012; Tsutsui, 2009; Tsutsui and Ubuka, 2012).

## IDENTIFICATION OF GnIH IN MAMMALS

In mammals, cDNAs that encode GnIH orthologs, LPXRFamide peptides, have been investigated by a gene database search (Hinuma et al., 2000; for a review, see Tsutsui and Ubuka, 2012). Mammalian LPXRFamide peptides are also designated as RFamide-related peptides (RFRP) from its structure. Although human, macaque, and bovine LPXRFamide precursor cDNAs encoded three RFRPs (RFRP-1, -2, and -3), only RFRP-1 and RFRP-3 possessed a C-terminal LPXRFamide (X = L or Q) motif, and RFRP-2 had C-terminal RSamide or RLamide sequences (**Figure 1**). On the other hand, rodents do not have RFRP-2 sequence in their precursors (see the precursor sequences of rat and hamster in **Figure 1**). Although the positions that encode GnIH-RP-1/RFRP-1 or GnIH/RFRP-2 in the precursor polypeptides were conserved between birds and mammals (only RFRP-1 in rodents), the positions of GnIH-RP-2 and RFRP-3 in their precursor polypeptides were different between birds and mammals (**Figure 1**).

The LPXRFamide motif at the C-terminus is followed by glycine as an amidation signal and arginine or lysine as endoproteolytic basic amino acids in mammals as well as birds (**Figure 1**). Endogenous LPXRFamide peptides can also be cleaved at basic amino acids at their N-termini. However, there were some exceptions in the cleavage site at the N-terminal, such as that of Siberian hamster RFRP-1 (**Figure 1**). Up until now, bovine RFRP-1 (Fukusumi et al., 2001) and -3 (Yoshida et al., 2003), rat RFRP-3 (Ukena et al., 2002), Siberian hamster RFRP-1 and -3 (Ubuka et al., 2012a), macaque RFRP-3 (Ubuka et al., 2009b), and human RFRP-1 and -3 (Ubuka et al., 2009c) are identified as mature peptides in mammals (**Figure 1**; for reviews, see Tsutsui and Ukena, 2006; Tsutsui et al., 2007, 2010a,b, 2012; Tsutsui, 2009; Tsutsui and Ubuka, 2012).

## GnIH RECEPTOR

**FIGURE 2:**
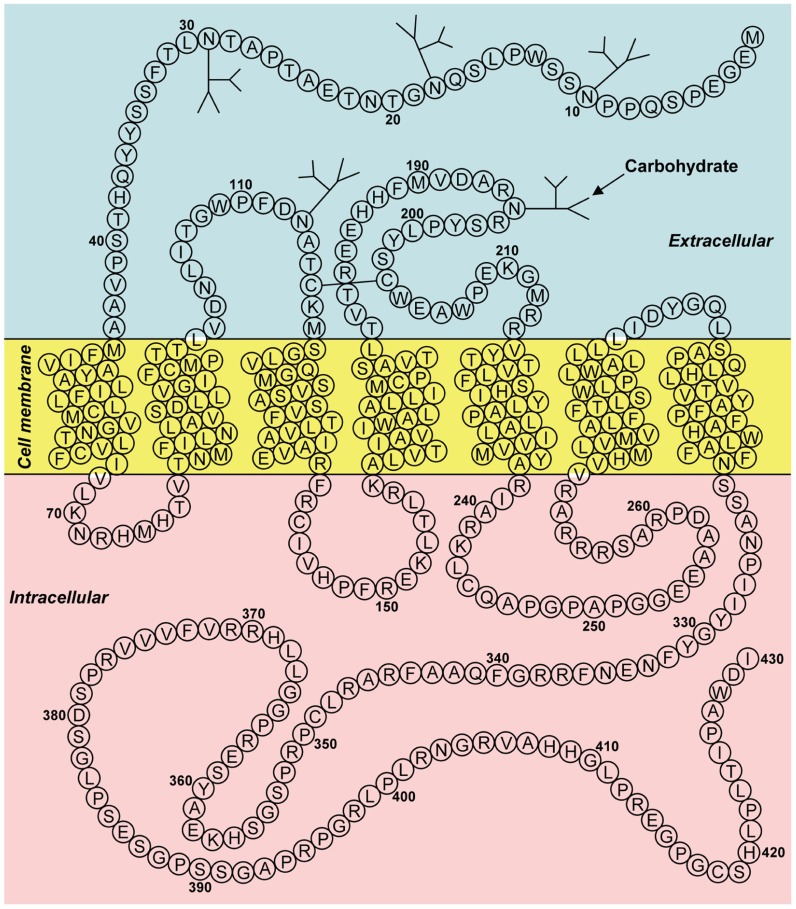
**Two-dimensional representation of the receptor (GPR147) for human GnIH (RFRP)**. The transmembrane region was predicted using SOSUI (Hirokawa et al., 1998). Glycosylation and disulfide bridge sites were predicted by GPCRDB (Horn et al., 1998). The accession number is AB040104. Adapted from Ubuka et al. (2008b).

Bonini et al. (2000) have identified two G protein-coupled receptor (GPCR) for neuropeptide FF (NPFF), and designated them as NPFF1 (identical to GPR147) and NPFF2 (identical to GPR74). Hinuma et al. (2000) have also reported a specific receptor for RFRP and named it OT7T022, which was identical to GPR147. The binding affinities for GPR147 and GPR74 and the efficacies on signal transduction pathway were examined, using various analogs of RFRPs and NPFF. RFRPs showed a higher affinity for GPR147, whereas NPFF had potent agonistic activity for GPR74 (Bonini et al., 2000; Liu et al., 2001). Taken together, GPR147 (NPFF1, OT7T022) was suggested to be the receptor for RFRP (mammalian GnIH). **Figure 2** shows the predicted two-dimensional structure of human GPR147 from its nucleotide sequence (AB040104; Ubuka et al., 2008b, 2009c). It was shown that RFRPs suppress the production of cAMP in ovarian cells of Chinese hamster transfected with GPR147 cDNA, suggesting that GPR147 couples to G_αi_ protein (Hinuma et al., 2000).

To elucidate the mode of action of GnIH in birds, Yin et al. (2005) have identified GnIH receptor (GnIH-R) in the quail diencephalon and characterized its expression and binding activity. First, a cDNA encoding a putative GnIH-R was cloned by a combination of 3′/5′ RACE using PCR primers designed from the sequence of the receptor for RFRPs (GPR147). The crude membrane fraction of COS-7 cells transfected with the putative GnIH-R cDNA specifically bound GnIH and GnIH-RPs in a concentration-dependent manner, indicating that GPR147 is GnIH-R (Yin et al., 2005). GnIH-R also bound with high affinities to GnIH, GnIH-RPs and RFRPs, which have LPXRFamide (X = L or Q) motif at their C-termini. In contrast, C-terminal non-amidated GnIH failed to bind the receptor. Accordingly, the C-terminal LPXRFamide (X = L or Q) motif seems to be critical for its binding to GnIH-R (Yin et al., 2005; for reviews, see Tsutsui and Ukena, 2006; Tsutsui et al., 2007, 2010a,b, 2012; Tsutsui, 2009; Tsutsui and Ubuka, 2012). It was suggested that there is no functional difference among GnIH and GnIH-RPs because GnIH-R bound GnIH and GnIH-RPs with similar affinities (Yin et al., 2005). However, further studies are required to investigate if GnIH and GnIH-RPs work additively or synergistically to achieve their effects on the cells that express GnIH-R.

## DISTRIBUTION OF GnIH CELLS AND FIBERS IN THE BRAIN

### LOCATION OF GnIH NEUNONS IN THE BRAIN

The location of GnIH precursor mRNA was first investigated by Southern blot analysis of the RT-PCR products of GnIH precursor cDNA. Within the samples from telencephalon, diencephalon, mesencephalon, and cerebellum, GnIH precursor mRNA was only expressed in the quail diencephalon (Satake et al., 2001). *In situ* hybridization for GnIH precursor mRNA further showed that cells expressing GnIH mRNA were clustered in the paraventricular nucleus (PVN) in the hypothalamus (Ukena et al., 2003). Immunohistochemistry using GnIH antibody has revealed that clusters of GnIH-ir neurons were expressed in the PVN in quail (Tsutsui et al., 2000; Ubuka et al., 2003). GnIH expressing cell bodies were also clustered in the PVN in other birds (Bentley et al., 2003; Osugi et al., 2004; Ubuka et al., 2008a).

In mammals, expression of precursor mRNA of RFRP (mammalian GnIH) was only detected in the dorsomedial hypothalamic area (DMH) in the mouse and hamster brains by *in situ* hybridization (Kriegsfeld et al., 2006; Ubuka et al., 2012a). In the rat brain, RFRP precursor mRNA was expressed in the periventricular nucleus (PerVN), and the portion between the dorsomedial nucleus (DMN) and the ventromedial nucleus (VMN) of the hypothalamus (Hinuma et al., 2000; Legagneux et al., 2009). The majority of RFRP mRNA expressing neuronal cell bodies were localized in the intermediate periventricular nucleus (IPe) of the hypothalamus in the macaque (Ubuka et al., 2009b), and in the DMN and PVN in the sheep (Clarke et al., 2008).

### GnIH INNERVATION IN THE BRAIN

Although a dense population of GnIH neuronal cell bodies was only found in the PVN in quail, GnIH-ir neuronal fibers were widely distributed in the diencephalic and mesencephalic regions (Ukena et al., 2003). Dense networks of GnIH-ir fibers were found in the ventral paleostriatum, septal area, preoptic area (POA), median eminence, optic tectum, and the dorsal motor nucleus of the vagus. GnIH-ir neuronal fibers were also widely distributed in the diencephalic and mesencephalic regions in European starlings (Ubuka et al., 2008a) and white-crowned sparrows (Ubuka et al., 2012b). Thus, it was hypothesized that GnIH may participate not only in the regulation of pituitary function, but also in behavioral and autonomic mechanisms in birds.

GnIH-ir neuronal fibers were also widely distributed in the diencephalic and mesencephalic regions in rodents. Dense GnIH-ir fibers were observed in the lateral septal nucleus, medial POA, amygdala, arcuate nucleus (ARC); moderate GnIH-ir fibers were observed in the paraventricular thalamic nucleus, the paraventricular hypothalamic nucleus, and the central gray in Siberian hamsters (Ubuka et al., 2012a). Dense GnIH-ir fibers were also observed in limbic and hypothalamic structures in rats (Johnson et al., 2007). Accordingly, it was hypothesized that GnIH may also participate in behavioral and autonomic mechanisms in mammals.

**FIGURE 3:**
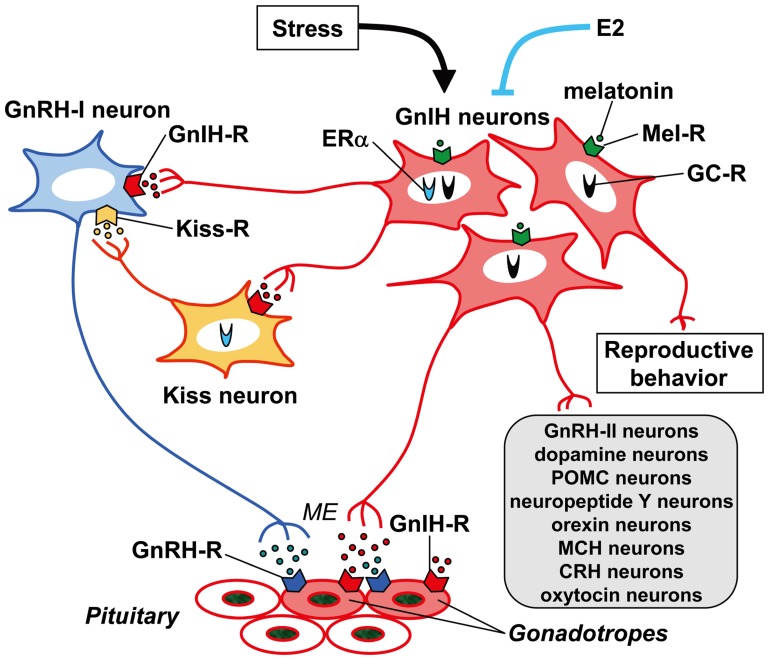
**Schematic model of GnIH (RFRP) action in the brain and pituitary**. GnIH (RFRP) neurons in the brain project their axons to GnRH-I neurons as well as to the median eminence (ME). GnIH receptor (GnIH-R; GPR147) is expressed on GnRH-I neurons as well as gonadotropes. GnIH may thus inhibit gonadotropin synthesis and release by inhibiting the activity of GnRH-I neurons as well as directly inhibiting the pituitary gonadotrope. GnIH (RFRP) neurons may also regulate GnRH-I neurons by regulating the activity of kisspeptin (Kiss) neurons that project to GnRH-I neurons. There are also reports showing that GnIH (RFRP) neurons project their axons to GnRH-II, dopamine, pro-opiomelanocortin (POMC), neuropeptide Y, orexin, melanin-concentrating hormone (MCH), corticotrophin-releasing hormone (CRH) and oxytocin neurons in the brain. GnIH (RFRP) inhibits reproductive behaviors of birds and mammals by possibly acting within the brain. The expression of GnIH (RFRP) is regulated by melatonin, stress, and estradiol-17β (E2). Expressions of melatonin receptor (Mel-R), glucocorticoid receptor (GC-R), or estrogen receptor α (ERα) in GnIH (RFRP) neurons were shown in several species. These mechanisms of action of GnIH (RFRP) on gonadotropin secretion or regulatory mechanism of GnIH (RFRP) expression may vary between species, sexes, and developmental stages.

Innervation of GnIH neuronal fibers was intensively investigated in the rhesus macaque brain (Ubuka et al., 2009b). Abundant GnIH-ir fibers were observed in the nucleus of the stria terminalis in the telencephalon; habenular nucleus, PVN of the thalamus, POA, PVN of the hypothalamus, IPe, ARC of hypothalamus, median eminence and dorsal hypothalamic area in the diencephalon; medial region of the superior colliculus, central gray substance of the midbrain and dorsal raphe nucleus in the midbrain; and parabrachial nucleus in the pons. GnIH-ir fibers were observed in close proximity to GnRH-I, dopamine, pro-opiomelanocortin, and GnRH-II neurons in the POA, IPe, ARC of hypothalamus, and central gray substance of midbrain, respectively. Qi et al. (2009) have shown that RFRP (mammalian GnIH) cells project to neuropeptide Y and pro-opiomelanocortin neurons in the ARC, orexin and melanin-concentrating hormone neurons in the lateral hypothalamic area, as well as orexin cells in the DMN and corticotrophin-releasing hormone and oxytocin cells in the PVN, GnRH neurons in the POA in sheep. GnIH neurons might thus regulate these important neural systems in addition to directly regulating pituitary gonadotropin release (Ubuka et al., 2009b; for reviews, see Tsutsui, 2009; Tsutsui et al., 2010a,b, 2012; Tsutsui and Ubuka, 2012; **Figure 3**).

## GnIH ACTION IN THE BRAIN

### MODULATION OF THE ACTIVITY OF GnRH AND KISSPEPTIN NEURONS BY GnIH

Immunohistochemical studies using light and confocal microscopy indicated that GnIH (RFRP)-ir axon terminals are in probable contact with GnRH neurons in birds (Bentley et al., 2003), rodents (Kriegsfeld et al., 2006; Ubuka et al., 2012a), monkeys (Ubuka et al., 2009b), and humans (Ubuka et al., 2009c). Thus, there is potential for the direct regulation of the activity of GnRH neurons by GnIH (RFRP) neurons (**Figure 3**).

Ubuka et al. (2008a) investigated the interaction of GnIH neuronal fibers and GnRH neurons in the European starling brain. It is generally accepted that birds possess at least two forms of GnRH in their brains. One form is GnRH-I which is thought to be released at the median eminence to stimulate the secretion of gonadotropins from the anterior pituitary (King and Millar, 1982; Miyamoto et al., 1982; Sharp et al., 1990; Ubuka and Bentley, 2009, 2011; Ubuka et al., 2009a). The second form of GnRH is GnRH-II (Miyamoto et al., 1984; Millar, 2003), which is thought to influence reproductive behaviors in birds (Maney et al., 1997) and mammals (Temple et al., 2003; Barnett et al., 2006). Double-label immunocytochemistry showed GnIH axon terminals on GnRH-I and GnRH-II neurons in the songbird brain (Bentley et al., 2003; Ubuka et al., 2008a). *In situ* hybridization of starling GnIH-R mRNA combined with GnRH immunocytochemistry further showed the expression of GnIH-R mRNA in GnRH-I and GnRH-II neurons (Ubuka et al., 2008a; **Figure 3**).

Double-label immunocytochemistry also showed GnIH axon terminals on GnRH neurons in the Siberian hamster brain (Ubuka et al., 2012a) and GPR147 was expressed in GnRH neurons (Ubuka et al., 2012a). Central administration of GnIH inhibits the release of gonadotropin in white-crowned sparrows (Bentley et al., 2006), Syrian hamsters (Kriegsfeld et al., 2006), rats (Johnson et al., 2007), and Siberian hamsters (Ubuka et al., 2012a) in a manner similar to peripheral administration of GnIH (Osugi et al., 2004; Kriegsfeld et al., 2006; Ubuka et al., 2006). Accordingly, GnIH may inhibit the secretion of gonadotropins by decreasing the activity of GnRH neurons in addition to directly regulating pituitary gonadotropin secretion (**Figure 3**).

Direct application of RFRP-3 to GnRH cells in cultured mouse brain slices decreased firing rate in a subpopulation of cells, further indicating a direct action of RFRP-3 on GnRH neurons (Ducret et al., 2009). In addition, RFRP-3 inhibited firing of kisspeptin-activated vGluT2 (vesicular glutamate transporter 2)-GnRH neurons as well as of kisspeptin-insensitive GnRH neurons (Wu et al., 2009). More recent data have confirmed a role for RFRP-3 using an antagonist, RF9, against GnIH-R. Central administration of RF9 to rats and mice led to marked increases in gonadotropin concentrations, providing a pronounced role of RFRP-3 as a key regulator of the reproductive axis in mammals (Pineda et al., 2010).

Recently, Rizwan et al. (2012) have investigated (1) whether RFRP-3 can directly inhibit LH secretion without inhibiting GnRH neurons; (2) whether RFRP-3 neurons project to GnRH neurons and rostral periventricular kisspeptin neurons in mice, and (3) whether GPR147 and GPR74 are expressed by these neurons. Intravenous treatment with the GPR147 antagonist RF9 increased plasma LH concentrations in castrated male rats but was unable to do so in the presence of the GnRH antagonist cetrorelix. Approximately 26% of GnRH neurons of male and diestrous female mice were apposed by RFRP-3 fibers, and 19% of kisspeptin neurons of proestrous female mice were apposed by RFRP-3 fibers. They further showed that 33% of GnRH neurons and 9–16% of rostral periventricular kisspeptin neurons expressed GPR147, whereas GPR74 was not expressed in either population. These data show that RFRP-3 can act on GnRH neurons as well as kisspeptin neurons to modulate reproduction in rodents (**Figure 3**).

### EFFECT OF CENTRAL ADMINISTRATION OF GnIH ON BEHAVIORS OF BIRDS AND MAMMALS

Central administration of GnIH or RFRP-3 to the third ventricle of the brain inhibited reproductive behavior of females in white-crowned sparrows (Bentley et al., 2006) or of males in rats (Johnson et al., 2007). It was known that GnRH-II enhances copulation solicitation in estrogen-primed female white-crowned sparrows exposed to the song of males (Maney et al., 1997). Because of the putative contact of GnIH neurons with GnRH-II neurons in white-crowned sparrows (Bentley et al., 2003), Bentley et al. (2006) investigated the effect of GnIH administration on copulation solicitation in females of this species. A centrally-administered physiological dose of GnIH inhibited copulation solicitation in estrogen-primed female white-crowned sparrows exposed to the song of males. Johnson et al. (2007) investigated the effect of central administration of RFRP-3 on reproductive behaviors of male rats. Behavioral tests indicated that RFRP-3 dose-dependently suppressed all facets of male sexual behavior. In contrast, immunoneutralization of RFRP in the rat brain increased male sexual behaviors. These results suggest that GnIH and RFRP inhibit reproductive behavior by inhibiting GnRH neuronal activities or by acting directly within the brain (**Figure 3**).

To identify the mechanism of GnIH neurons in the regulation of behavior, Ubuka et al. (2012b) investigated the effect of RNA interference (RNAi) of the GnIH gene on the behavior of white-crowned sparrows, a highly social songbird species. Administration of small interfering RNA against GnIH precursor mRNA into the third ventricle of male and female birds reduced resting time, spontaneous production of complex vocalizations, and stimulated brief agonistic vocalizations. GnIH RNAi further enhanced song production of short duration in male birds when they were challenged by playbacks of novel male songs. These behaviors resembled those of breeding birds during territorial defense. The overall results suggest that GnIH gene silencing induces arousal. In addition, the activities of male and female birds were negatively correlated with GnIH mRNA expression in the PVN. Density of GnIH neuronal fibers in the ventral tegmental area was decreased by GnIH RNAi treatment in female birds, and the number of GnRH neurons that received close appositions of GnIH neuronal fiber terminals was negatively correlated with the activity of male birds. In summary, GnIH may decrease arousal level resulting in the inhibition of specific motivated behavior, such as in reproductive contexts (Ubuka et al., 2012b).

Central administrations of GnIH or RFRP-3 can also stimulate feeding behavior in chicken or rats (Tachibana et al., 2005; Johnson et al., 2007; Murakami et al., 2008). There is a recent report showing that central administration of RFRP-3 can stimulate adrenocorticotropic hormone and oxytocin release, and induce anxiety behavior in rats (Kaewwongse et al., 2011). The fact that RFRP-ir fibers project to various neurons in the brain, such as dopamine and/or pro-opiomelanocortin neurons in the rat, sheep, and macaque (Qi et al., 2009; Ubuka et al., 2009b; **Figure 3**) suggests multiple functions of GnIH or RFRP in the brain.

## GnIH ACTION IN THE PITUITARY

Dense population of GnIH-ir fibers at the median eminence (ME) in quail (Tsutsui et al., 2000; Ukena et al., 2003) as well as in other birds (Bentley et al., 2003; Osugi et al., 2004; Ubuka et al., 2008a), suggested a direct action of GnIH in the regulation of pituitary function in birds (**Figure 3**). The fact that GnIH inhibits synthesis and/or release of gonadotropins from cultured quail and chicken anterior pituitary gland provides strong support for this function (Tsutsui et al., 2000; Ciccone et al., 2004). Peripheral administration of GnIH also inhibits gonadotropin synthesis and/or release in birds (Osugi et al., 2004; Ubuka et al., 2006). In mammals, abundant RFRP-ir fibers were observed in the ME of sheep (Clarke et al., 2008), macaque (Ubuka et al., 2009b), and humans (Ubuka et al., 2009c). As GnIH in birds, RFRP-3 inhibits gonadotropin synthesis and/or release from cultured pituitaries in sheep (Sari et al., 2009) and cattle (Kadokawa et al., 2009). Peripheral administration of RFRP-3 also inhibits gonadotropin release in sheep (Clarke et al., 2008), rats (Murakami et al., 2008), and cattle (Kadokawa et al., 2009). It was further shown that GnIH-R (GPR147) mRNA is expressed in gonadotropes in the human pituitary (Ubuka et al., 2009c). Taken together, it is likely that GnIH and RFRP-3 directly act on the pituitary to inhibit gonadotropin secretion from the pituitary at least in these avian and mammalian species (**Figure 3**).

Recently, Smith et al. (2012) measured the concentration of RFRP-3 in hypophyseal portal blood in ewes during the non-breeding (anestrous) season and during the luteal and follicular phases of the estrous cycle in the breeding season. Pulsatile RFRP-3 secretion was observed in the portal blood of all animals. Mean RFRP-3 pulse amplitude and pulse frequency were higher during the non-breeding season. RFRP-3 was virtually undetectable in peripheral blood plasma. To determine the role of secreted RFRP-3, Smith et al. (2012) further examined its effects on GnRH-stimulated LH secretion in hypothalamo–pituitary-disconnected ewes, and a significant reduction in the LH response to GnRH was observed by RFRP-3 administration. These data show that RFRP-3 is secreted into portal blood to act on pituitary gonadotropes, reducing the action of GnRH in sheep (Smith et al., 2012).

On the contrary it was suggested that GnIH or RFRP-3 may not act directly on the pituitary in some birds and rodents, because there are relatively few or no GnIH (RFRP)-ir fibers in the ME of Rufous-winged sparrows (Small et al., 2007), hamsters (Kriegsfeld et al., 2006; Ubuka et al., 2012a), and rats (Rizwan et al., 2009). Rizwan et al. (2009) have injected a retrograde tracer Fluoro-Gold intraperitoneally in rats. The majority of GnRH neurons were labeled but essentially no RFRP neurons were labeled. In contrast, intracerebral injections of Fluoro-Gold into the rostral POA resulted in the labeling of 75 ±5% of RFRP cell bodies. These observations suggested that RFRP-3 is not a hypophysiotropic neuroendocrine hormone in rats (Rizwan et al., 2009). However, there are also studies indicating that RFRP-3 can act directly to inhibit gonadotropin release from the pituitary of rats (Murakami et al., 2008). More extensive studies, analyzing peptide release into the hypophyseal portal blood, fiber projections by retrograde labeling, etc., are needed to elucidate the hypophysiotropic action of GnIH in various animals (for reviews, see Tsutsui et al., 2007, 2010a,b, 2012; Tsutsui, 2009; Tsutsui and Ubuka, 2012).

Since GPR147 couples to G_αi_ to inhibit adenylyl cyclase (AC; Hinuma et al., 2000), GPR147 (GnIH-R) activation may reduce intracellular cAMP levels, and reduce the activities of cAMP-dependent protein kinase (PKA) and mitogen-activated protein kinase (MAPK) signaling cascade. On the other hand, GnRH receptor (GnRH-R) couples with G_αq/11_ to activate phospholipase C (PLC), which results in the production of inositol tri-phosphate (IP3) and diacylglycerol (DAG). In turn, IP3 and DAG increase intracellular Ca^2^^+^ and activate the protein kinase C (PKC) pathway and MAPK signaling. GnRH-R was also reported to be coupled to G_αs_ to stimulate AC/cAMP/PKA pathway. A recent study using immortalized mouse gonadotrope cell line (LβT2 cells) has demonstrated that the inhibitory action of mouse RFRPs on gonadotropin gene expression is mediated by an inhibition of AC/cAMP/PKA-dependent extracellular signal-regulated kinase (ERK) pathway (Son et al., 2012). In the sheep, RFRP-3 can inhibit both GnRH-induced intracellular Ca^2^^+^ increase and ERK phosphorylation, impacting GnRH-induced gonadotropin release and synthesis (Sari et al., 2009). In the chicken, activation of GnRH-R can activate AC and stimulate cAMP responsive element (CRE) binding protein. GnIH may partly inhibit this GnRH-induced CRE activation, thus impacting gene transcription (Shimizu and Bédécarrats, 2010).

## REGULATION OF GnIH EXPRESSION

### EFFECT OF MELATONIN

Identification of the regulatory mechanisms governing GnIH expression is important in understanding the physiological role of the GnIH system. Photoperiodic mammals rely on the annual cycle of changes in nocturnal secretion of melatonin to drive their reproductive responses (Bronson, 1990). In contrast, a dogma has existed that birds do not use seasonal changes in melatonin secretion to time their reproductive effort, and a role for melatonin in birds has remained enigmatic (Wilson, 1991; Juss et al., 1993). Despite the accepted dogma, there is strong evidence that melatonin is involved in the regulation of several seasonal processes, including gonadal activity and gonadotropin secretion (Ohta et al., 1989; Guyomarc’h et al., 2001; Rozenboim et al., 2002). Ubuka et al. (2005) hypothesized that melatonin may be involved in the induction of GnIH expression, thus influencing gonadal activity. The action of melatonin on the expression of GnIH was studied in quail, a highly photoperiodic bird species. Because the pineal gland and eyes are the major sources of melatonin in the quail (Underwood et al., 1984), Ubuka et al. (2005) analyzed the effects of pinealectomy (Px) combined with orbital enucleation (Ex; Px plus Ex) on the expression of GnIH precursor mRNA and GnIH peptide. Subsequently, melatonin was administered to Px plus Ex birds. Px plus Ex decreased the expression of GnIH precursor mRNA and the content of mature GnIH peptide in the hypothalamus. Melatonin administration to Px plus Ex birds caused a dose-dependent increase in the expression of GnIH precursor mRNA and the production of mature peptide. They also investigated the expression of melatonin receptor in GnIH neurons. *In situ* hybridization combined with immunocytochemistry for GnIH revealed that the mRNA of Mel1c, a melatonin receptor subtype, was expressed in GnIH-ir neurons in the PVN. Autoradiography of melatonin receptors further revealed specific binding of melatonin in the PVN. Accordingly, melatonin appears to act directly on GnIH neurons through its receptor to induce expression of GnIH (Ubuka et al., 2005; **Figure 3**).

Chowdhury et al. (2010) further investigated the role of melatonin in the regulation of GnIH release and the negative correlation of GnIH release with LH release in quail. Melatonin administration dose-dependently increased GnIH release from hypothalamic explants *in vitro*. A clear diurnal change in GnIH release was observed in quail, and this change was negatively correlated with changes in plasma LH concentrations. GnIH release during the dark period was greater than that during the light period in explants from quail exposed to long-day (LD) photoperiods. Conversely, plasma LH concentrations decreased during the dark period. In contrast to LD, GnIH release increased under short-day (SD) photoperiods, when the duration of nocturnal secretion of melatonin increases. These results indicate that melatonin may play a role in stimulating not only GnIH expression but also GnIH release, thus inhibiting plasma LH concentrations in quail (**Figure 3**).

A similar, but opposite, action of melatonin on the inhibition of the expression of RFRP was shown in Syrian and Siberian hamsters, both photoperiodic mammals (Revel et al., 2008; Mason et al., 2010; Ubuka et al., 2012a). The level of RFRP mRNA and the number of RFRP-ir cell bodies were reduced in sexually quiescent Syrian and Siberian hamsters acclimated to SD photoperiod, compared to sexually active animals maintained under LD photoperiod. The photoperiodic variation of RFRP expression was abolished in Px hamsters and injections of LD hamsters with melatonin reduced the expression of RFRP down to SD levels, indicating a dependence upon melatonin (Revel et al., 2008; Ubuka et al., 2012a). There are also reports showing that the expression of RFRP precursor mRNA is regulated by melatonin in sheep (Dardente et al., 2008) and rats (Gingerich et al., 2009). These results demonstrate that the expression of GnIH and RFRP is photoperiodically modulated via a melatonin-dependent process (**Figure 3**).

Quail is a LD breeder, which activates its reproductive activity in LD and suppresses its reproductive activity in SD. It is understandable that the expression of GnIH is stimulated by a nocturnal hormone melatonin and SD when the duration of melatonin secretion is long. Accordingly, it was hypothesized that the increase of GnIH expression may inhibit reproductive activity in SD in quail (Ubuka et al., 2005). The opposite but similar thoughts can be applied to sheep. Sheep is a SD breeder that activates its reproductive activity in SD and suppresses its reproductive activity in LD. It is also understandable that the expression of RFRP is inhibited by a nocturnal hormone melatonin and SD when the duration of melatonin secretion is long. Accordingly, it was hypothesized that the increase of RFRP expression may inhibit reproductive activity in LD in sheep (Dardente et al., 2008). On the other hand, it was difficult to understand the inhibitory effect of melatonin or SD on RFRP expression in hamsters, because hamsters are LD breeders (Revel et al., 2008; Mason et al., 2010). However, recent report has shown that RFRP may have a stimulatory effect on gonadotropin secretion in SD in Siberian hamsters (Ubuka et al., 2012a). Long duration of melatonin secretion in SD may need to decrease RFRP expression to inhibit reproductive activities of Siberian hamsters in SD. Another recent work in male Syrian hamsters suggested that GnIH might be excitatory in LD as well (Ancel et al., 2012). Long duration of melatonin secretion in SD may decrease RFRP expression to inhibit reproductive activities and short duration of melatonin secretion in LD may increase RFRP expression to stimulate reproductive activities of Syrian hamsters in LD (Ancel et al., 2012). Further studies are required to understand the role of melatonin controlling GnIH and RFRP expression in seasonal breeders.

### EFFECT OF STRESS

Stress leads to reproductive dysfunction in many species, including rodents and humans. Calisi et al. (2008) hypothesized that stress effects upon reproduction are mediated via the hypothalamic GnIH system in birds. They examined the effects of capture-handling stress in the hypothalamus of male and female adult house sparrows. There were more GnIH-positive neurons in fall birds versus those sampled in spring, and a significant increase in GnIH-positive neurons was seen in stressed birds in spring. These data imply an influence of stress upon the GnIH system that changes over the annual cycle of reproduction (**Figure 3**).

Kirby et al. (2009) showed that both acute and chronic immobilization stress lead to an up-regulation of the expression of RFRP in the DMH of adult male rats and that this increase in RFRP is associated with inhibition of downstream HPG activity. They also showed that adrenalectomy blocks the stress-induced increase in RFRP expression. Immunohistochemistry revealed that 53% of RFRP cells express receptors for glucocorticoids (GCs), suggesting that adrenal GCs can mediate the stress effect through direct action on RFRP cells. These data show that stress-induced increases in adrenal GCs cause an increase in RFRP that contributes to hypothalamic suppression of reproductive function (**Figure 3**).

Papargiris et al. (2011) tested if GnIH (RFRP) mediates the inhibitory effect of stress on LH secretion in ovariectomized ewes using a psychosocial stressor, isolation/restraint. Isolation/restraint stress increased plasma cortisol concentrations and decreased plasma LH concentrations. However, there was no significant effect of stress on RFRP peptide or mRNA levels, with no difference between control or stressed ewes. Furthermore, there was no difference in the number of RFRP-ir cells double-labeled for Fos between control and stressed ewes and there was no difference in the cellular expression of RFRP mRNA between groups. Accordingly, GnIH (RFRP) may not mediate the effects of stress on LH secretion in ewes or the effect of stress may depend on the presence of gonadal sex steroids.

### EFFECT OF SEX STEROIDS

Estrogen secreted by the ovary feedbacks to the brain and pituitary to regulate gonadotropin secretion (Herbison, 1998; Petersen et al., 2003). Wintermantel et al. (2006) found that estrogen positive feedback to generate the preovulatory gonadotropin surge was normal in estrogen receptor (ER) β knockout mice, but absent in ERα mutant mice. Because GnRH neurons do not express ERα, estrogen positive feedback upon GnRH neurons must be indirect. RFRP (mammalian GnIH) neuronal system may be involved in estrogen feedback signaling to GnRH neurons because RFRP neurons in rodents express ERα and respond with c-Fos expression to an acute administration of estradiol-17β (E2; Kriegsfeld et al., 2006; **Figure 3**).

The role of RFRP-3 in regulating ovulatory function was investigated in female hamsters (Gibson et al., 2008). The cellular activity of RFRP neurons was suppressed at the time of the LH surge, suggesting removal of negative feedback by RFRP-3 at this time. The SCN, the master circadian clock triggering ovulation in rodents, projects to a large proportion of RFRP neurons, providing a mechanism for timing removal of negative drive on the GnRH system. Activities of the SCN, GnRH, and RFRP neurons were coordinated with ovulation (Gibson et al., 2008). Li et al. (2012) investigated the expression patterns in the reproductive axis of the female pig across the estrous cycle. The hypothalamic levels of both RFRP and its receptor mRNA were lowest in estrus and peaked in the proestrus and diestrus phases. Smith et al. (2010) examined Kiss1 and RFRP mRNA throughout the menstrual cycle of a female primate, rhesus macaque. Kiss1-expressing cells were found in the POA and ARC, and RFRP-expressing cells were located in the PVN/DMN. Kiss1 expression in the caudal ARC and POA was higher in the late follicular phase of the cycle (just before the GnRH/LH surge) than in the luteal phase. RFRP expression was also higher in the late follicular phase suggesting that RFRP fine tunes GnRH/LH surge in primates. There are also reports showing the correlation of RFRP expression and testicular activities of male mice (Sethi et al., 2010a,b).

Molnár et al. (2011) investigated the possibility that RFRP neurons are involved in estrogen feedback signaling to the reproductive axis in mice. They compared the expression of RFRP mRNA of ovariectomized mice, with and without E2 replacement. Subcutaneous administration of E2 via silastic capsules for 4 days significantly down-regulated RFRP mRNA expression. In ovariectomized mice, low levels of ERα immunoreactivity were detectable in 18.7 ± 3.8% of RFRP neurons, whereas RFRP neurons did not exhibit ERβ immunoreactivity. The estrogenic down-regulation of RFRP expression may contribute to estrogen positive feedback to the reproductive axis. However, whether E2 regulates RFRP neurons directly or indirectly remains an open question because ERα immunoreactivity is present only in a subset of RFRP cells (**Figure 3**).

Poling et al. (2012) examined changes in RFRP neurons in mice of both sexes during development and under different adulthood hormonal milieus. They identified two interspersed subpopulations of RFRP cells (high RFRP-expressing, HE; low RFRP-expressing, LE), which have unique developmental and steroidal regulation characteristics. The number of LE cells robustly decreased during postnatal development, whereas HE cell number increased significantly before puberty. In adults, they found that E2 and testosterone moderately repress RFRP expression in both HE and LE cells, whereas the non-aromatizable androgen dihydrotestosterone has no effect. They determined that approximately 25% of RFRP neurons coexpress ERα in each sex, whereas RFRP cells do not express androgen receptor in either sex, regardless of hormonal milieu. They detected coexpression of GPR147 but no coexpression of GPR74 in GnRH neurons of both intact and gonadectomized males and females. RFRP-3 may thus exert its effects on reproduction either directly, via GPR147 in a subset of GnRH neurons, and/or indirectly, via upstream regulators of GnRH (**Figure 3**).

## RECENT PROGRESS IN GnIH STUDIES ON FISH AND HUMANS

We have described the progress of GnIH research investigating its function in the brain and pituitary of birds and mammals. Recently, there were important progresses in the study of GnIH peptides in fish and humans as we briefly summarize them below.

A cDNA encoding three GnIH orthologs, LPXRFamide peptides, was cloned from the goldfish brain by a combination of 3′/5′ RACE (Sawada et al., 2002). Mass spectrometric analyses revealed that a tridecapeptide (SGTGLSATLPQRFamide) is expressed in the brain as an endogenous ligand. Immunoreactive cell bodies were restricted to the nucleus posterioris periventricularis and the nervus terminalis and immunoreactive fibers were distributed in several brain regions including the nucleus lateralis tuberis pars posterioris and pituitary (Sawada et al., 2002). Amano et al. (2006) analyzed the hypophysiotropic activity of the three goldfish LPXRFamide peptides (gfLPXRFa-1, -2, and -3) in sockeye salmon. gfLPXRFa-ir cell bodies were detected in the nucleus posterioris periventricularis of the hypothalamus and immunoreactive fibers were distributed in various brain regions and the pituitary in sockeye salmon. gfLPXRFamide peptides stimulated the release of FSH, LH, and GH from cultured pituitary cells (Amano et al., 2006). In contrast, Zhang et al. (2010) have identified the orthologous GnIH genes in zebrafish, stickleback, medaka, and Takifugu. The zebrafish GnIH precursor contained three putative LPXRFamide peptides. Intraperitoneal administration of the mature zebrafish LPXRFa-3 (zfLPXRFa-3) significantly reduced the basal serum LH level in goldfish (Zhang et al., 2010).

Moussavi et al. (2012) examined the effects of synthetic gfLPXRFamide peptides on pituitary LHβ and FSHβ subunit, and gfLPXRFamide peptide receptor (gfLPXRFa-R) mRNA levels and LH secretion in goldfish. Intraperitoneal injections of gfLPXRFa-3 increased pituitary LHβ and FSHβ mRNA levels at early to late gonadal recrudescence, but reduced serum LH and pituitary gfLPXRFa-R mRNA levels, respectively, at early to mid-recrudescence and later stages of recrudescence. Static incubation with gfLPXRFa-3 elevated LH secretion from dispersed pituitary cell cultures from prespawning fish, but not at other recrudescent stages. gfLPXRFa-3 suppressed LHβ mRNA levels at early recrudescence and prespawning but elevated LHβ at mid-recrudescence, and consistently attenuated FSHβ mRNA in a dose-specific manner *in vitro*. These results indicate that the effect of gfLPXRFa-3 depends on maturational status and administration route in goldfish (Moussavi et al., 2012).

Recently, the mature peptide structures of human GnIH peptides (human RFRP-1 and RFRP-3) were identified by mass spectrometry (Ubuka et al., 2009c). Because the structure of human RFRP-3 was identical to the structure of sheep RFRP-3, physiological functions of human RFRP-3 were studied in the sheep. It was shown that sheep/human RFRP-3 inhibits gonadotropin synthesis and release *in vitro* (Sari et al., 2009) and gonadotropin release *in vivo* (Clarke et al., 2008; Smith et al., 2012). In view of its potent inhibition of gonadotropin secretion, human RFRP-3 (human GnIH) has the potential of an alternative or adjunct therapeutic agent to inhibit endogenous levels of gonadotropins and steroid hormones in humans. Thus, RFRP has therapeutic potential in the treatment of hormone-dependent diseases, such as precocious puberty, endometriosis, uterine fibroids, benign prostatic hyperplasia, and prostatic and breast cancers.

## Conflict of Interest Statement:

The authors declare that the research was conducted in the absence of any commercial or financial relationships that could be construed as a potential conflict of interest.
